# Impaired age self-consciousness in latent schizophrenia

**DOI:** 10.1192/j.eurpsy.2021.1277

**Published:** 2021-08-13

**Authors:** R. Suleimanov, M. Artemieva, I. Danilin, A. Lazukova, V. Sokolov

**Affiliations:** Psychiatry And Medical Psychology, RUDN University, Moscow, Russian Federation

**Keywords:** Latent Schizophrenia, impaired age self-consciousness

## Abstract

**Introduction:**

The topic of research was phenomenon of impaired age self-consciousness in non-psychotic latent schizophrenia patients defined.

**Objectives:**

To explore features of impaired age self-identity and to determine syndromic affiliation of the syndrome in comparison with premorbid personality disorders traits.

**Methods:**

The study sample comprised 141 patients with latent schizophrenia (pseudo neurotic (F21.3 - 64.5%, 91 patients), coenesthopathic (F20.8 - 25.5%, 36 patients) and pseudo psychopathic (F21.4, - 9.9%, 14 patients)) aged 16-31 (average 22.1 years old) in 2007-2019. A follow-up, experimental psychological and clinical study was conducted.

**Results:**

The onset of impaired age self-identity was dominated by a radical drop of the subjective age in self-conscious mind of the patients accompanied by a tormented feeling of loss of self-dependence, role autonomy, helplessness, inability of decision making and to be answerable. Patients described this sudden condition as a loss of ‘maturity feeling’ and return to the juvenile perception of self. In a delusive and unclear manner, phrases such as ‘I feel inferior to others as if a helpless child among adults’, ‘I feel as if my childhood is back’ were uttered. Excessive worrying and enlivening of childhood memories were also included. This correlates to occurrence of humble and sometimes dependent/avoidant behavior, feeling of helplessness and fear with respect to caring for one self, rising subordination and suggestibility.

**Conclusions:**

This phenomenon of regress to earlier ontogenetic level of personal development reported as impaired age self-consciousness can thus be regarded as an obligate form of depersonalization in patients with latent schizophrenia.

